# Supplementary material for: Cohesin couples transcriptional bursting probabilities of inducible enhancers and promoters
Source: Nat Commun. 2022 Jul 27;13:4342. doi: 10.1038/s41467-022-31192-9 (PMC9329429; doi:10.1038/s41467-022-31192-9)
Supplement: Supplementary file 1 — Supplementary Information [file 41467_2022_31192_MOESM1_ESM.pdf]

Supplementary Information

Cohesin couples transcriptional bursting probabilities of inducible enhancers and promoters

Irene Robles-Rebollo, Sergi Cuartero, Adria Canellas, Sarah Wells, Mohammad M Karimi, Elisabetta Mereu, Alexandra G Chivu, Holger Heyn, Chad Whilding, Dirk Dormann, Samuel Marguerat, Inmaculada Rioja, Rab K Prinjha, Michael PH Stumpf, Amanda G Fisher, Matthias Merkenschlager

Supplementary Figures

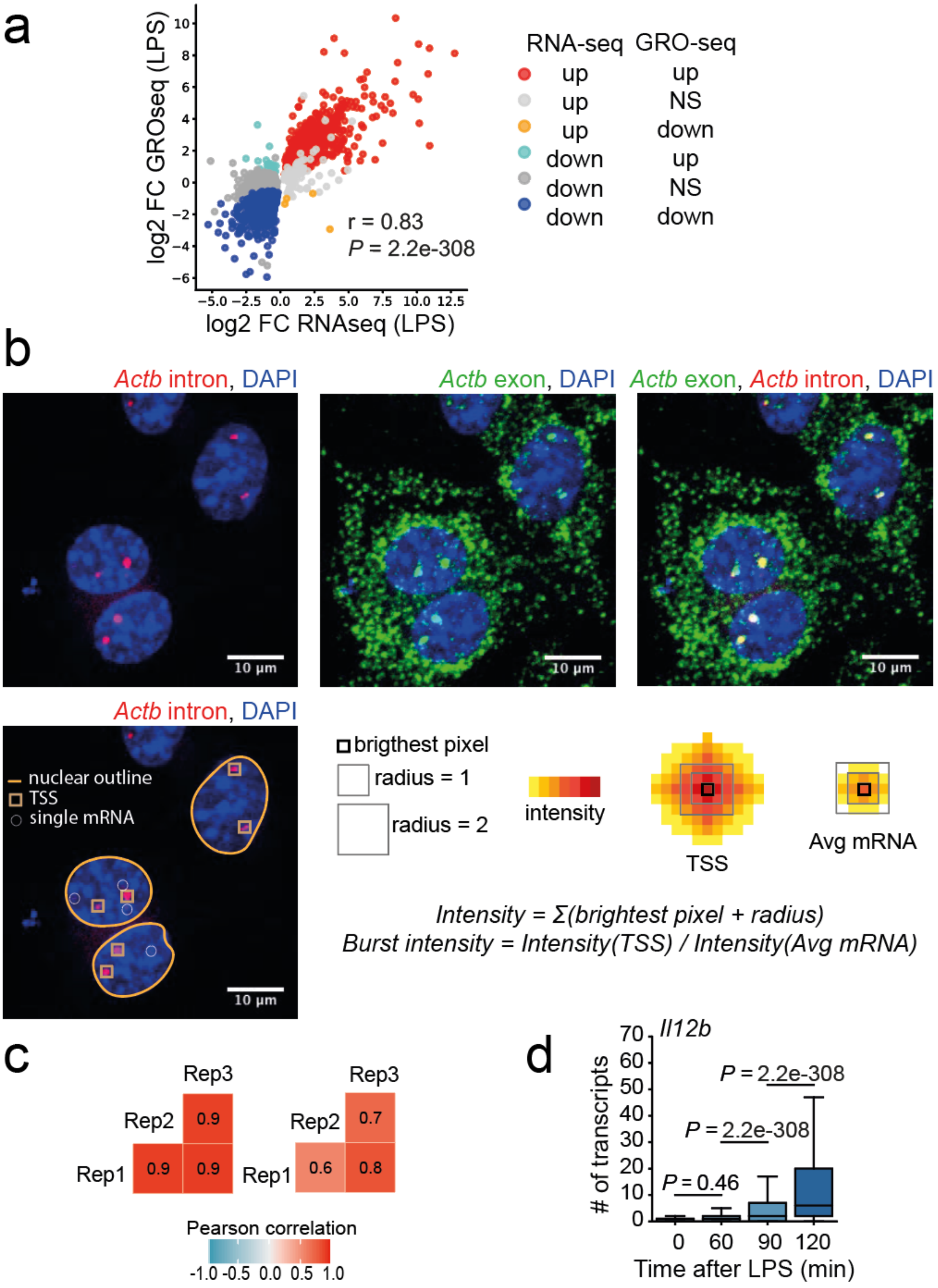

Supplementary Figure 1. smRNA-FISH approach and validation.

a) Correlation between log_2 fold changes in gene expression between untreated and LPS-treated macrophages by RNA-seq (2h LPS versus untreated) and GRO-seq (1h LPS versus untreated). NS: not significant. r = Pearson correlation. Two-sided Pearson's product-moment correlation, null hypothesis: correlation = 0. Three independent biological replicates for RNA-seq, 2 independent biological replicates for GRO-seq.

b) Identification of transcription start sites and burst measurements illustrated using *Actb* intron and exon smRNA-FISH as an example. Nuclear outlines identified by CellProfiler, transcription start sites and individual transcripts identified by FISHQuant. Mature macrophages are quiescent diploid cells, and up to two bursts for each probe are assigned per nucleus. Burst intensity is determined as the integrated intensity of the brightest pixel plus a radius of 1 or 2 pixels divided by the integrated intensity of the same area of the averaged individual mRNA image.

c) Correlation between smRNA-FISH replicates. Set 1: *Il12b, Stat1, Actb*: 3 independent biological replicates with a total of 8899 cells (left), set 2: *Cd40, Tnf, Atf3*: 3 independent biological replicates, n = 12372 cells (right).

d) Accumulation of transcript copy numbers of inducible genes with time after LPS activation. P values determined by Tukey HSD test (ANOVA design formula ~ Sample + Replicate), 2-sided, and adjusted for multiple testing. N = 24160 cells, 4 independent biological replicates.

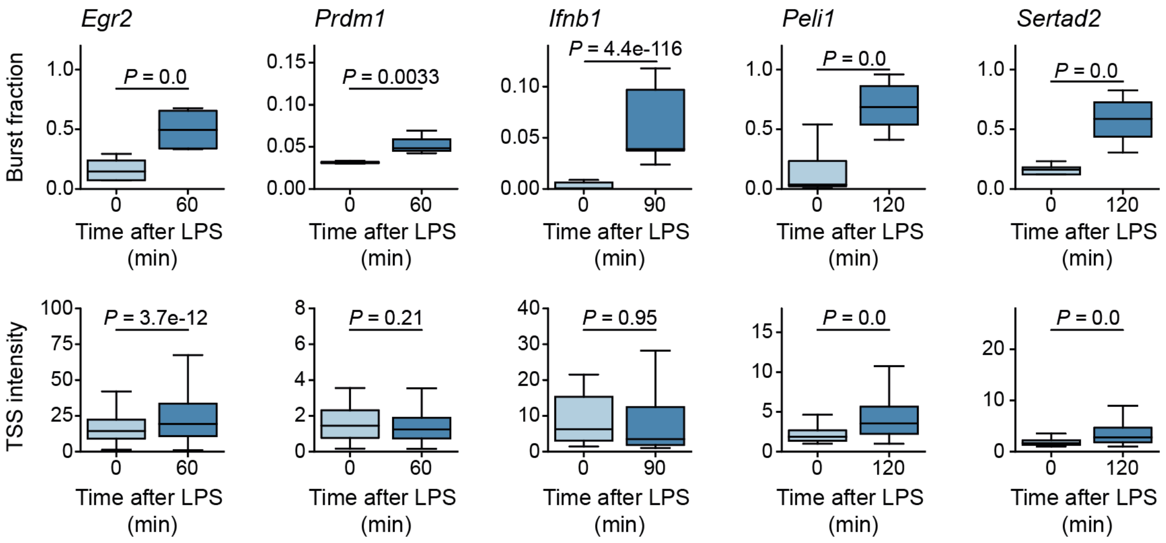

Supplementary Figure 2. Inducible expression of *Egr2, Prdm1, Ifnb1, Peli1* and *Sertad2* is regulated primarily by the fraction of actively transcribing alleles

Box plots of smRNA-FISH measurements of burst fraction (top) and burst intensity (bottom). N = 68286 cells, 3-7 independent biological replicates per transcript.

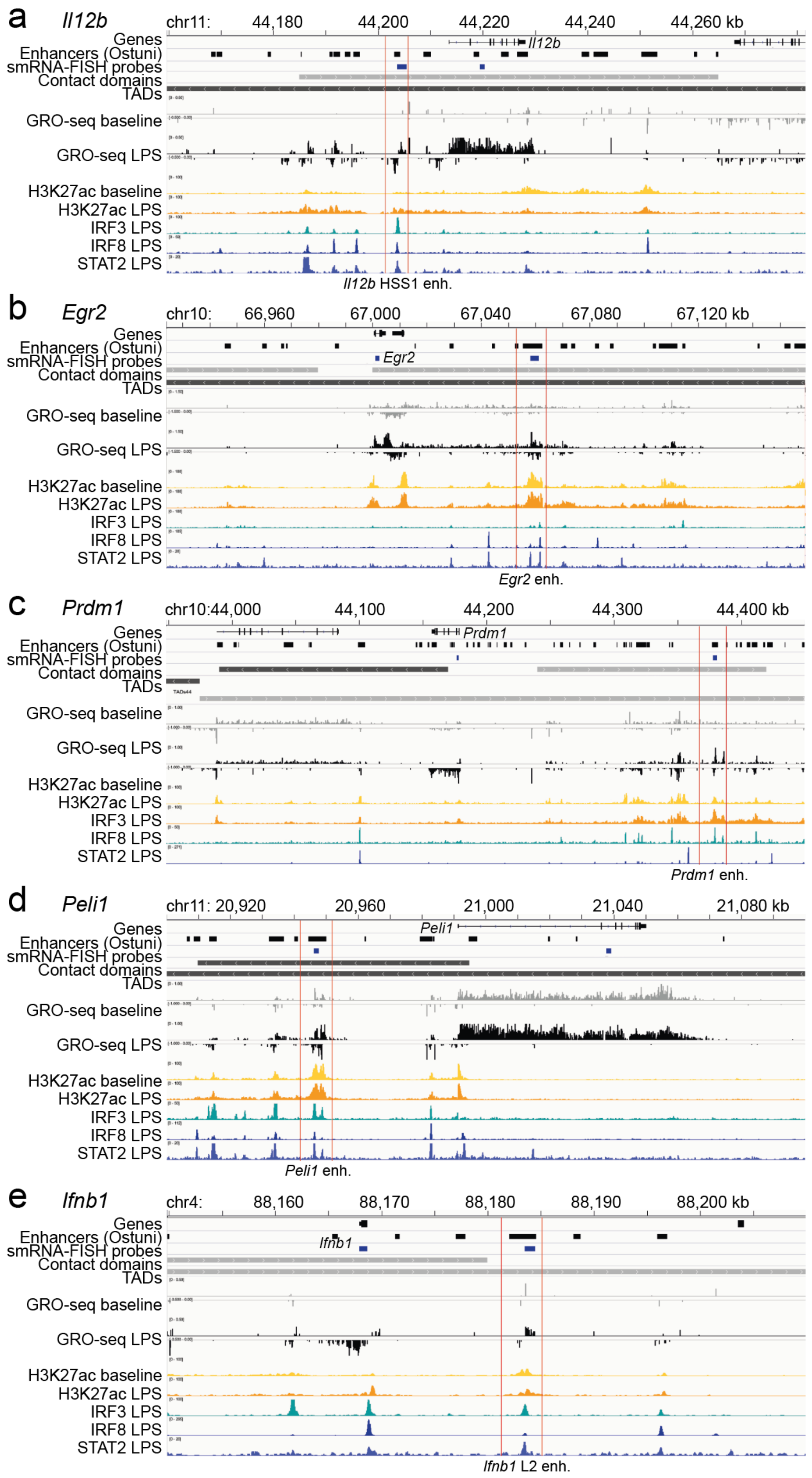

Supplementary Figure 3. Inducible enhancers associated with smRNA-FISH target genes.

We identified enhancers[31] that are both detectable by GRO-seq and show significant change between baseline and 1h LPS treatment of wild-type macrophages. This analysis identified 1112 inducibly transcribed enhancers in wild-type macrophages. Of these inducible enhancers, 1048 remained intact and 64 were downregulated (adj $P < 0.05$) in *Rad21*$^{-/-}$ macrophages. Inducible enhancers located in the same TADs as smRNA-FISH target genes remain largely intact in cohesin-deficient macrophages. The Figure shows IGV screenshots of the TADs containing *Il12b, Egr2, Prdm1, Peli1* and *Ifnb1*. Intergenic macrophage enhancers are indicated[31]. GRO-seq transcribed, LPS-inducible enhancers (adj. $P < 0.05$ at 1h LPS activation) are shown. Enhancers with reduced GRO-seq signal in *Rad21*$^{-/-}$ macrophages are marked in red (adj. $P < 0.05$ at 1h LPS activation).

a) *Il12b*: other expressed genes in TAD: *Ublcp1, Rnf145*. Intergenic transcribed enhancers: 9, of 3 respond to LPS in 1h GRO-seq. None are found deregulated by GRO-seq in *Rad21*$^{-/-}$ macrophages at 1h LPS.

b) *Egr2*: other expressed genes in TAD: *Ado, Gm10797*. Intergenic transcribed enhancers: 21, of which 6 respond to LPS in 1h GRO-seq. One is found downregulated by GRO-seq in *Rad21*$^{-/-}$ macrophages at 1h LPS.

c) *Prdm1*: other expressed genes in TAD: *Atg5, Prep*. Intergenic transcribed enhancers: 26, of which 7 respond to LPS in 1h GRO-seq. Four are found downregulated by GRO-seq in *Rad21*$^{-/-}$ macrophages at 1h LPS.

d) *Peli1*: other expressed genes in TAD: *Vps54, Ugp2, 2610024D14Rik, Gm12042*. Intergenic transcribed enhancers: 12, of which 4 respond to LPS in 1h GRO-seq. None are found deregulated by GRO-seq in *Rad21*$^{-/-}$ macrophages at 1h LPS.

e) *Ifnb1*: other expressed genes in TAD: *Mllt3, BC057079, Ptplad2, Ifna2, Klhl9, Gm13283, Ifna6, Gm17476*. Intergenic transcribed enhancers: 7, of which none respond to LPS in 1h GRO-seq.

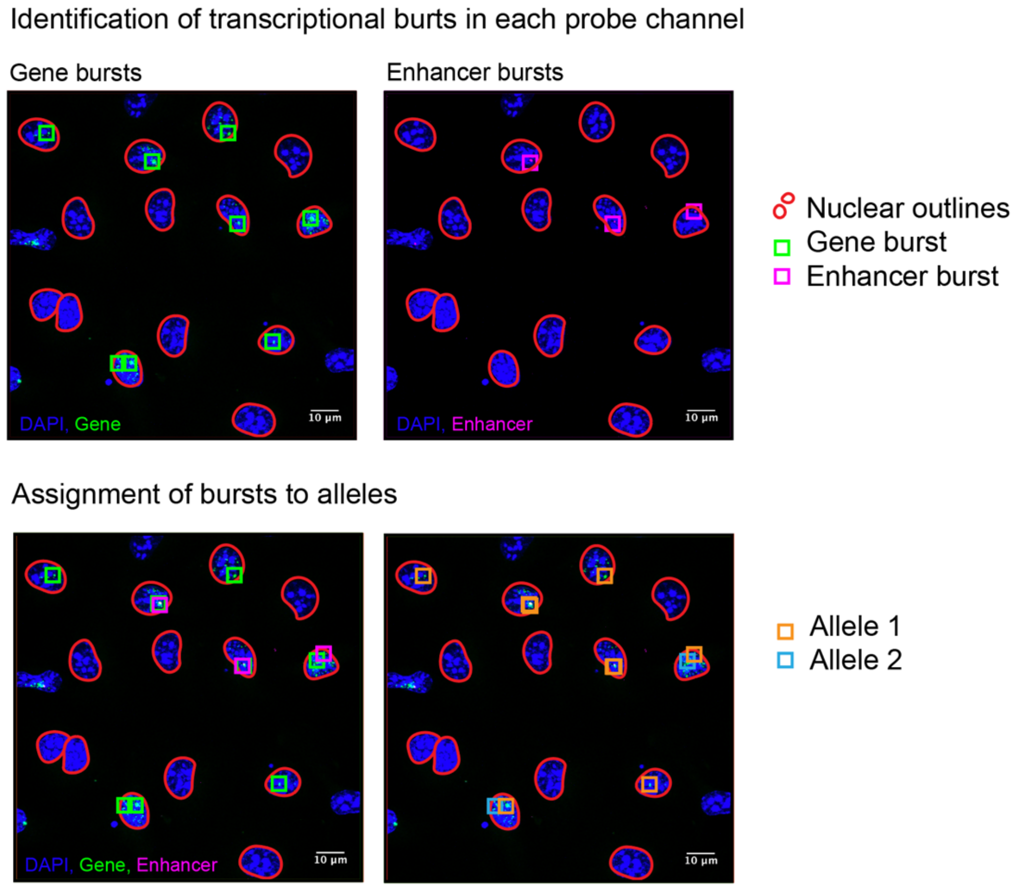

Supplementary Figure 4. Assigning bursts of enhancers and genes to alleles.

Burst outlines are identified by FISH-quant from each probe channel. Up to two bursts for each probe are assigned per nucleus. For each nucleus, distances between the brightest pixel of gene and enhancer bursts are measured. Burst pairs are assigned to alleles based on proximity. A burst can be assigned only to one allele, and one allele of a gene can only be assigned one burst at any one time. The genomic distances between genes and enhancers analysed here are limited: 61kb for the *Egr2* enhancer and TSS, 198kb for the *Prdm1* enhancer and TSS, 10kb for the *Il12b* HSS1 and *Il12b* TSS, 14kb for *Ifnb1* L2 and the *Ifnb* TSS, and 43kb for the *Peli1* enhancer and TSS. Based on data shown in Fig. 3f, enhancer and gene burst are assigned to the same allele if they are < 1 μm apart.

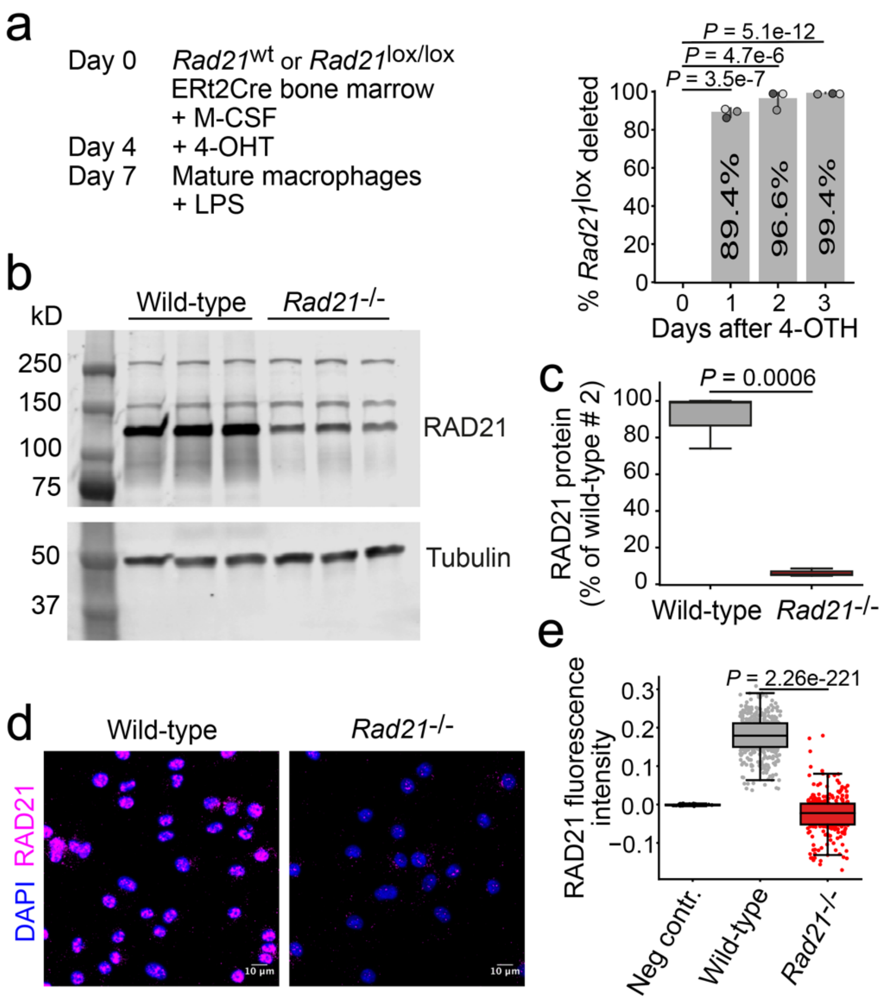

Supplementary Figure 5. Inducible RAD21 depletion

a) Outline of experiment and 4-OHT-induced deletion of floxed *Rad21* (*Rad21*^{lox}) loci assayed by quantitative genomic PCR. Negative control *Rad21*$^{lox/lox}$ macrophages without ERt2Cre induction by 4-OHT. Statistical test: One way ANOVA. Three independent biological replicates. Error bars : 95% confidence interval.

b) Western blot of RAD21 and Tubulin in wild-type and *Rad21*$^{-/-}$ macrophages. Molecular weight markers are on the left.

c) Quantification of western blot in b). Percentage of RAD21 expression relative to wild-type sample # 2. Two-sided equal variance t-test. Three independent biological replicates.

d) Example images of RAD21 immunofluorescence staining. Representative of 3 independent biological replicates, n = 752 cells.

e) Immunofluorescence analysis of RAD21 protein levels in wild-type and *Rad21*$^{-/-}$ macrophages. RAD21 levels are the mean intensity in the nucleus minus the mean intensity in the cytoplasm. Negative control: Immunofluorescence analysis without primary antibody. Statistical test: equal variance t-test. Three independent biological replicates, n = 752 cells.

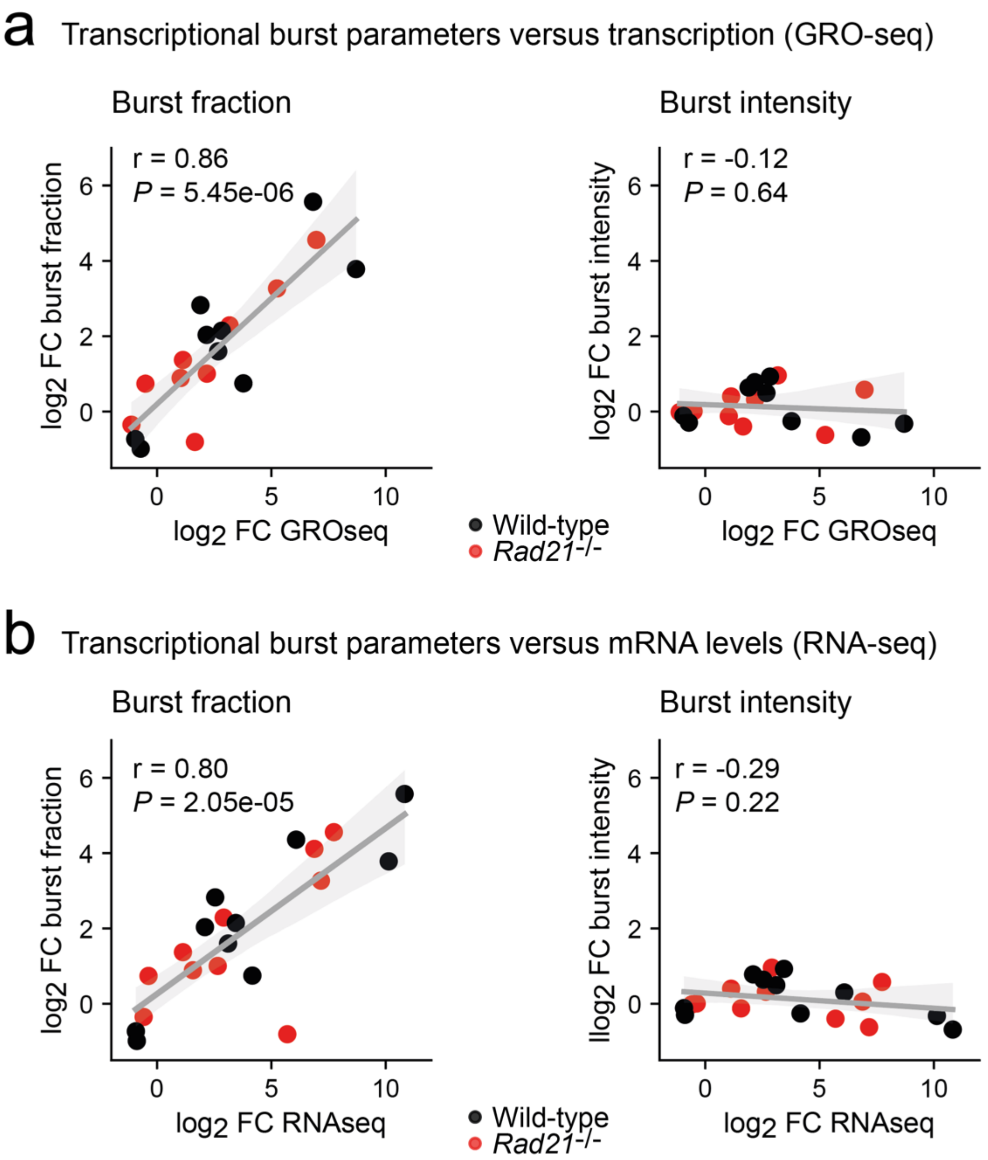

Supplementary Figure 6. Differences in transcript abundance between *Rad21*-deficient and wild-type macrophages are best explained by transcriptional burst fraction

a) Transcriptional burst parameters versus transcriptional activity as assessed by GRO-seq. r = Pearson correlation. Two-sided Pearson's product-moment correlation. Null hypothesis: correlation = 0. Two GRO-seq replicates. N = 227969 cells, 3-8 independent biological replicates per transcript. Error bands: 95% confidence interval.

b) Transcriptional burst parameters versus mRNA levels as assessed by RNA-seq exon counts. r = Pearson correlation. Statistical test: Pearson's product-moment correlation. Null hypothesis: correlation = 0. Three RNA-seq replicates. N = 227969 cells, 3-8 independent biological replicates per transcript. Error bands: 95% confidence interval.

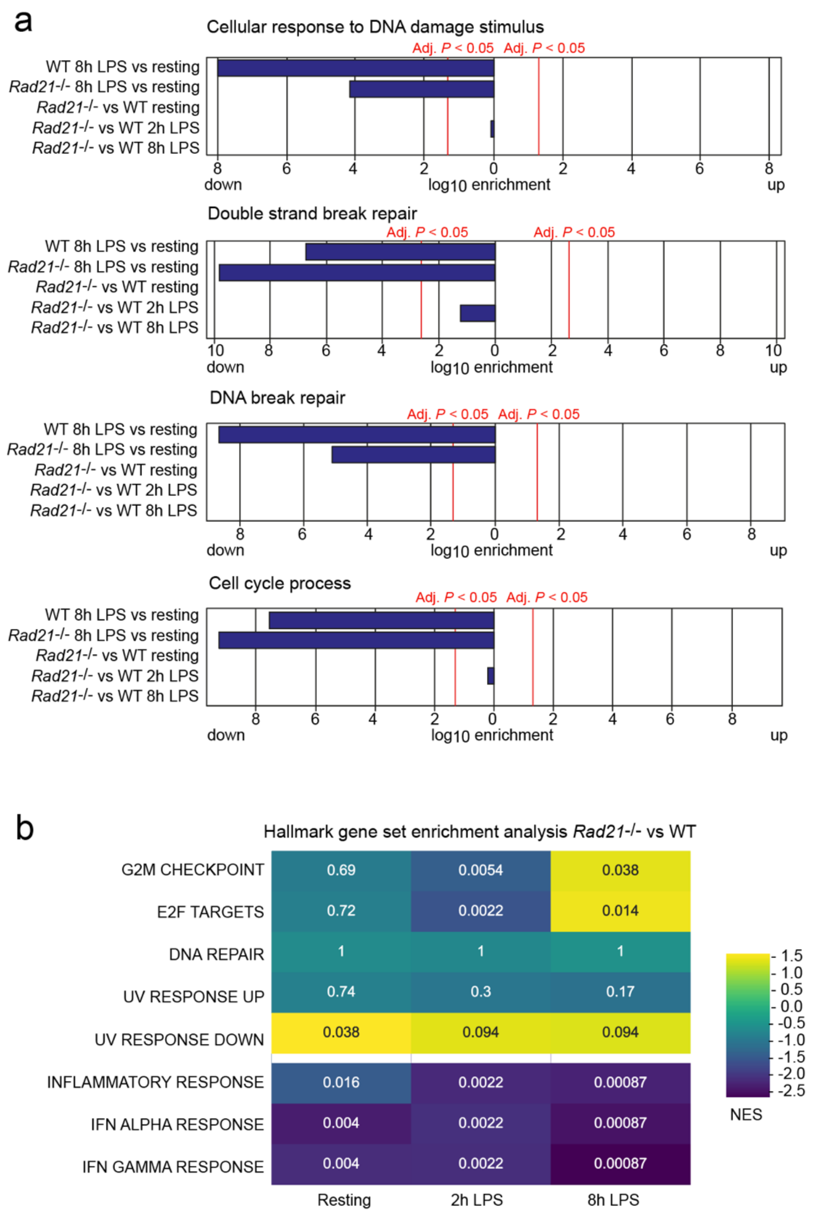

Supplementary Figure 7. No evidence for activation of cell cycle or DNA damage pathways in *Rad21*-deficient macrophages

a) Enrichment of the indicated gene ontology terms among up- (right) or downregulated genes (left) in response to LPS activation and *Rad21* deletion in macrophages. Note the downregulation of genes related to DNA damage, DNA repair and the cell cycle in response to LPS activation in both wild-type and *Rad21*$^{-/-}$ macrophages. There were no significant differences in the enrichment of the gene ontology terms related to DNA damage, DNA repair or the cell cycle between wild-type and *Rad21*$^{-/-}$ macrophages in resting or LPS-activated conditions. Red lines indicate statistical significance (adj. P < 0.05). Three independent biological RNA-seq replicates per genotype and condition.

b) Hallmark gene set enrichment analysis in *Rad21*$^{-/-}$ versus wild-type macrophages. NES = normalised enrichment score. Three independent biological RNA-seq replicates per genotype and condition.

Supplementary Table 1. smRNA-FISH probes

Ref. number	Excitation	Probe	GeneSymbol	Type
VB4-10432	488	*Actb*	*Actb*	Exon
VB1-20973	550	*Actb* intron	*Actb*	Intron
VB6-15721	650	*Atf3*	*Atf3*	Exon
VB1-15088	550	*Cd40*	*Cd40*	Exon
VB6-10663	650	*Cxcl10*	*Cxcl10*	Exon
VPTZ792	650	*Egr2* Enh	*Egr2*	eRNA
VPRWEN4	550	*Egr2*	*Egr2*	Intron
VPMFWXN	650	*Peli1* Enh	*Peli1*	Erna
VPNKRGW	488	*Fh1* intron	*Fh1*	Intron
VPMFWWY	550	*Hprt* intron	*Hprt*	Intron
VPPRJ37	550	*Il12b* HSS1	*Il12b*	eRNA
VB1-12995-VC	550	*Ifit1*	*Ifit1*	Exon
VB4-12687-VC	488	*Ifnb1*	*Ifnb1*	Exon
VB6-20182	650	*Il12b*	*Il12b*	Exon
VB4-20975	488	*Il12b* intron	*Il12b*	Intron
VPNKRJA	650	*Ifnb1* L2	*Ifnb1*	eRNA
VB6-3205469	650	*Peli1*	*Peli1*	Exon
VB1-3035672-VC	550	*Peli1* intron	*Peli1*	Intron
VB4-6000742	488	*Peli1* intron	*Peli1*	Intron
VPWCWEW	650	*Prdm1* Enh	*Prdm1*	eRNA
VPU62UY	550	*Prdm1* intron	*Prdm1*	Intron
VB6-3204369-VC	650	*Sertad2*	*Sertad2*	Exon
VPDJXFM	550	*Sertad2* intron	*Sertad2*	Intron
VB4-6000742	488	*Spred2*	*Spred2*	Exon
VB6-6000741	650	*Spred2* intron	*Spred2*	Intron
VB1-11676	550	*Stat1*	*Stat1*	Exon
VB4-18211	488	*Tnf*	*Tnf*	Exon
