# Supplementary material for: Cohesin couples transcriptional bursting probabilities of inducible enhancers and promoters
Source: Nat Commun. 2022 Jul 27;13:4342. doi: 10.1038/s41467-022-31192-9 (PMC9329429; doi:10.1038/s41467-022-31192-9)
Supplement: Supplementary file 3 — Reporting Summary [file 41467_2022_31192_MOESM3_ESM.pdf]

Corresponding author(s): M Merkenschlager

Last updated by author(s): Apr 26, 2022

Reporting Summary

Nature Portfolio wishes to improve the reproducibility of the work that we publish. This form provides structure for consistency and transparency in reporting. For further information on Nature Portfolio policies, see our Editorial Policies and the Editorial Policy Checklist.

Statistics

For all statistical analyses, confirm that the following items are present in the figure legend, table legend, main text, or Methods section.

n/a	Confirmed	
☐	☒	The exact sample size (*n*) for each experimental group/condition, given as a discrete number and unit of measurement
☐	☒	A statement on whether measurements were taken from distinct samples or whether the same sample was measured repeatedly
☐	☒	The statistical test(s) used AND whether they are one- or two-sided<br>*Only common tests should be described solely by name; describe more complex techniques in the Methods section.*
☐	☒	A description of all covariates tested
☐	☒	A description of any assumptions or corrections, such as tests of normality and adjustment for multiple comparisons
☐	☒	A full description of the statistical parameters including central tendency (e.g. means) or other basic estimates (e.g. regression coefficient) AND variation (e.g. standard deviation) or associated estimates of uncertainty (e.g. confidence intervals)
☐	☒	For null hypothesis testing, the test statistic (e.g. *F*, *t*, *r*) with confidence intervals, effect sizes, degrees of freedom and *P* value noted<br>*Give P values as exact values whenever suitable.*
☒	☐	For Bayesian analysis, information on the choice of priors and Markov chain Monte Carlo settings
☒	☐	For hierarchical and complex designs, identification of the appropriate level for tests and full reporting of outcomes
☐	☒	Estimates of effect sizes (e.g. Cohen's *d*, Pearson's *r*), indicating how they were calculated

Our web collection on statistics for biologists contains articles on many of the points above.

Software and code

Policy information about availability of computer code

Data collection	Inmunofluorescence images were analysed using CellProfiler v2.2.0.<br>smFISH images in coverslips were analysed using CellProfiler v1.0.0 in MATLAB 2014a with specific image-based transcriptomics modules (Battich et al. 2013, Stoeger et al. 2015) for nuclei, cytoplasm and dot identification.<br>smFISH images in chamber slides were analysed using and CellProfiler v2.2.0 to obtain cell and nuclei outlines, and FISH-QUANT v3a to identify Transcription Start Sites (TSSs) and single dots. TSSs were quantified and assigned to alleles based on distance using a custom Julia script (Julia v1.6, https://github.com/IreneRobles/TSSs).<br>100bp paired end RNASeq reads were aligned to mouse genome mm9 using Tophat2 v2.0.10 (Kim et al, 2013) with arguments "--library-type fr-first strand --b2-very-sensitive --b2-L 25" with gene annotation from Ensembl version 67. Read counts on genes were summarised using HTSeq-count v0.5.3p9 (Anders et al., 2014). RUVseq Bioconductor v1.18.0 package was applied with RUVs function and k=3 (Risso et al. 2014) for ERCC spike-ins and replicate samples as controls.<br>GRO-Seq libraries were aligned to mouse genome mm9 using bowtie with arguments '-l 30 -m 10 -n 2 –trim3 10'. Read counts on enhancers (Otsuni et al., 2013) were computed using summarizeOverlaps function from GeomicAlignments R Package.<br>scRNAseq libraries were mapped with the RNA pipeline of the GEMTools 1.7.0 suite (Marco-Sola et al. 2012) using default parameters (6% of mismatches, minimum of 80% matched bases, and minimum quality threshold of 26) and the genome references for human (Gencode release 24, assembly GRCh38.p5), mouse (Gencode release M8, assembly GRCm38.p4), and dog (Ensembl v84, assembly CanFam3.1).
Data analysis	Differential expression analysis was performed in R (v 4.1) using then, differential expression analysis was performed using DESeq2 (Love et al. 2014). Pearson correlation tests were done with the cor.test function in R. To test significant changes in event frequency across replicates we used a Cochran-Mantel-Haenszel test with replicate as strata variable in R (v 4.1). Bonferroni correction for multiple testing was implemented in MutipleTesting.jl Julia package in Julia (v 1.6). To test significant changes in distribution values across replicates, we used one way ANOVA with Tukey HSD correction in R (v 4.1). To test whether the relationship between two variables differs between conditions we

implemented 2-way ANOVA in R (v 4.1). https://github.com/IreneRobles/Code_Paper contains all code required to produce the figures.

For manuscripts utilizing custom algorithms or software that are central to the research but not yet described in published literature, software must be made available to editors and reviewers. We strongly encourage code deposition in a community repository (e.g. GitHub). See the Nature Portfolio guidelines for submitting code & software for further information.

Data

Policy information about availability of data

All manuscripts must include a data availability statement. This statement should provide the following information, where applicable:

- Accession codes, unique identifiers, or web links for publicly available datasets
- A description of any restrictions on data availability
- For clinical datasets or third party data, please ensure that the statement adheres to our policy

The image data generated in this study have been deposited in the EMBL-EBI BioImage Archive database under accession code S-BIAD338 [https://www.ebi.ac.uk/biostudies/BioImages/studies/S-BIAD338].

The scRNA-seq data generated in this study have been deposited in the Gene Expression Omnibus (GEO) database under accession code GSE190622 [https://www.ncbi.nlm.nih.gov/geo/query/acc.cgi?acc=GSE190622].

The GRO-seq, RNA-seq, H3K27ac and ChIP-seq data used in this study are available in the GEO database under accession code GSE108599 [https://www.ncbi.nlm.nih.gov/geo/query/acc.cgi?acc=GSE108599].

The macrophage Hi-C data used in this study are available in the GEO database under accession code GSE115524 [https://www.ncbi.nlm.nih.gov/geo/query/acc.cgi?acc=GSE115524].

The PU.1 ChIP-seq data used in this study are available in the GEO database under accession code GSE56121 [https://www.ncbi.nlm.nih.gov/geo/query/acc.cgi?acc=GSE56121].

The STAT2 ChIP-seq data used in this study are available in the GEO database under accession code GSE56123 [https://www.ncbi.nlm.nih.gov/geo/query/acc.cgi?acc=GSE56123].

The IRF3 ChIP-seq data used in this study are available in the GEO database under accession code GSE67343 [https://www.ncbi.nlm.nih.gov/geo/query/acc.cgi?acc=GSE67343].

The processed data for every figure generated in this study are provided in the Supplementary Information/Source Data file.

Field-specific reporting

Please select the one below that is the best fit for your research. If you are not sure, read the appropriate sections before making your selection.

☒ Life sciences ☐ Behavioural & social sciences ☐ Ecological, evolutionary & environmental sciences

For a reference copy of the document with all sections, see nature.com/documents/nr-reporting-summary-flat.pdf

Life sciences study design

All studies must disclose on these points even when the disclosure is negative.

Sample size	No statistical test was used to determine sample size. Three or more biological replicates per group (as indicated in the figure legends) were used to provide sufficient data to allow statistical inference. We imaged as many cells as we could in 8 samples overnight.
Data exclusions	RNAseq: No data were excluded except for standard quality control filtering during sequencing analysis. This includes filtering genes with low read counts due to their low statistical power using a DESeq2 independent filtering approach. Standard filtering also involves removing reads aligning to multiple positions. Some smFISH samples were excluded due to high background or to low cell numbers as long as there were at least 3 replicates per sample.
Replication	Number of replicates and independent experiments detailed in figure legends, a minimum of three biological replicates were used for each experiment.
Randomization	No randomization was required. Mice used for primary macrophage culture were chosen according to genotype.
Blinding	Investigators were not blinded to group allocation. We considered that blinding was not relevant to this study because we used quantitative assays for all experiments and the computational framework was identical for all samples and replicates

Reporting for specific materials, systems and methods

We require information from authors about some types of materials, experimental systems and methods used in many studies. Here, indicate whether each material, system or method listed is relevant to your study. If you are not sure if a list item applies to your research, read the appropriate section before selecting a response.

Materials & experimental systems

n/a	Involved in the study	
☐	☒	Antibodies
☒	☐	Eukaryotic cell lines
☒	☐	Palaeontology and archaeology
☐	☒	Animals and other organisms
☒	☐	Human research participants
☒	☐	Clinical data
☒	☐	Dual use research of concern

Methods

n/a	Involved in the study	
☒	☐	ChIP-seq
☒	☐	Flow cytometry
☒	☐	MRI-based neuroimaging

Antibodies

Antibodies used	goat a-rabbit 488 antibody (Invitrogen, A11034)<br>goat a-rabbit 680 (A21109, Life Technologies)<br>goat a-mouse 680 (A21057, Life Technologies)<br>Rad21 (Abcam, 154769)<br>Tubulin (Sigma, T9026)
Validation	According to the manufacturer's website a-Rad21 (Abcam, 154769) is suitable for western-blot (1/1000) and immunofluorescence (1:500 dilution). Goat a-rabbit 488 antibody (Invitrogen, A11034) was used for immunofluorescence and a negative control where no primary antibody was added was used to assess the secondary antibody background. a-Tubulin (Sigma, T9026) was suitable for immunoblotting according to the manufacturer's webpage at a dilution of 1:1,000 which says ("The antibody is specific for α-tubulin in immunoblotting assays and may be used for localization of α-tubulin in cultured cells or tissue sections"). Goat a-rabbit 680 (A21109, Life Technologies) and goat a-mouse 680 (A21057, Life Technologies) were used at a 1:10,000 dilution. According to the manufacturer's webpage, for both antibodies "the sensitivity and specificity of each lot is confirmed using Western blot".

Animals and other organisms

Policy information about studies involving animals; ARRIVE guidelines recommended for reporting animal research

Laboratory animals	Rosa26-ERt2Cre Rad21WT/WT or Rad21lox/lox mice on a mixed C57BL/6 129 background were maintained under SPF conditions with a 12h light/dark cycle and food and water ad libitum. Male and female mice between 6 weeks and 6 months of age were used for experiments.
Wild animals	The study did not involve wild animals.
Field-collected samples	The study did not involve samples collected from the field.
Ethics oversight	Mouse work was performed according to the Animals (Scientific Procedures) Act Mouse work was done under a project licence issued by the UK Home Office, UK following review by the Imperial College London Animal Welfare and Ethical Review Body (AWERB).

Note that full information on the approval of the study protocol must also be provided in the manuscript.
