# Supplementary figures and images for: Cohesin couples transcriptional bursting probabilities of inducible enhancers and promoters
Source: Nat Commun. 2022 Jul 27;13:4342. doi: 10.1038/s41467-022-31192-9 (PMC9329429; doi:10.1038/s41467-022-31192-9)

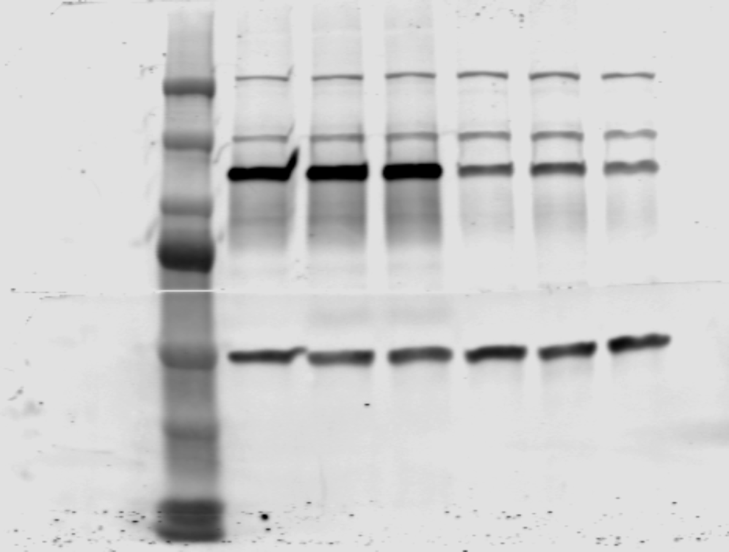

Supplement: Supplementary file 4 — Source Data [file 41467_2022_31192_MOESM4_ESM.zip › SourceData/SupFig5_WB.png]
